# Case Report: Severe Hypernatremia From Ingestion of One's Own Urine

**DOI:** 10.3389/fmed.2022.929180

**Published:** 2022-06-15

**Authors:** Waye Hann Kang, N. A. Kamaruddin, Norlela Sukor

**Affiliations:** ^1^Department of Medicine, Faculty of Medicine and Health Sciences, University Tunku Abdul Rahman, Kuala Lumpur, Malaysia; ^2^Department of Medicine, Faculty of Medicine, Universiti Kebangsaan Malaysia Medical Centre, Kuala Lumpur, Malaysia

**Keywords:** hypernatremia, urophagia, urine ingestion, electrolytes, osmotic diuresis

## Abstract

An often unrecognized cause of hypernatremia is the ingestion of fluids or substances with high osmolality. We hereby report a case of severe hypernatremia with acute kidney injury in a severely debilitated patient with acute gouty arthritis who resorted to ingesting his own urine. Hypernatremia induced by drinking urine could be attributed to many underlying mechanisms, one of the important possible causes is the resultant high serum urea that leads to significant osmotic diuresis and a further increase in free water clearance. To the best of our knowledge this is the first case report that describes this unique cause of hypernatremia.

## Introduction

Hypernatremia is defined as abnormally high serum sodium concentration and usually is a sequela of increased water loss, reduced water intake and rarely increased ingestion of salt or salt-related products. An often forgotten cause is those induced by osmotic diuresis from ingestion of fluids or substances with high osmolality.

## Case Description

A 40-year-old man with an underlying Eisenmenger's Syndrome due to an inoperable atrial septal defect (ASD) was brought to the emergency department for severe flare of his gouty arthritis. As a result of this severe debilitating joint pains and swellings over his knees, toes, fingers and elbows the patient was completely incapacitated and confined to his bed. He has been staying alone and hence had no access to food and water, thus had to resort to drinking his own urine for the past 5 days until he was found by his relatives who brought him to seek medical attention.

Patient denied any history of vomiting or diarrhea. He claimed he still had minimal urine output throughout the 5 days. He defaulted his cardiology and rheumatology consultations and denied taking any traditional or over-the-counter medications.

Clinically he was severely dehydrated and lethargic with a full Glasgow coma scale of 15. His heart rate was slightly tachycardic at 104 beats per min, whilst his blood pressure was stable at 110/60 mm Hg. There were marked tenderness over his elbows, knees, and toes with restricted range of movements.

He was diagnosed to have acute gouty arthritis with acute kidney injury and hypernatremia secondary to dehydration. There was no urine output during his initial presentation. Intravenous crystalloid was promptly administered (see Table 1). The patient was initially started on 550cc of normal saline (NS) over 5 h. However, the drip regime was changed to 140cc of quarter saline dextrose 5% (QSD5%) over 3 h when the serum sodium results came back as 160 mmol/L. With this drip regime his serum sodium levels gradually reduced to 152 mmol/L, hence his drip regime was further adjusted. The patient was subsequently given a total of 350cc half saline (HS) over 6 h to slow down the rate of sodium decrement.

**Table 1:** Biochemical parameters from day of admission till day 1 of hospital stay.

**Parameters**	**Normal range**	**2016**	**D0**	**D1**					
			**19:00 h**	**00:00 h**	**03:00 h**	**09:00 h**	**17:00 h**	**21:00 h**	**23:00 h**
Haematocrit	40.1–50.6%	49.3	55.3					49.3	55.3
Hemoglobin	13.5–17.4 g/dL	15.9	17.7					15.9	17.7
Na	135–145 mmol/L	140	160	152	150	150	146	149	148
K	3.5–4.5 mmol/L	2.9	4.2	4.0			3.9	3.9	3.7
Urea	3.2–7.4 mmol/L	3.0	48	46.9			45.4	41.5	42.6
Creatinine	63.6–110.5 umol/L	56.6	267.6	271.3			267.7		203.4
Albumin	35–50 g/L	29	26						
Serum Osmolarity	mOsm/L						343		340
Urine Osmolarity	mOsm/L						542		576
Urine sodium	mmol/L						45		40
Uric acid	mmol/L		1,136						
Fluids			550 cc NS	140 cc QSD5%	350 cc HS	200 cc NS	200 cc S3%	Stop IV fluids	
Daily Urine output				150cc					
Daily fluid balance				+1350cc					

*NS, normal saline; QSD5%, quarter saline dextrose 5%; HS, half saline; S3%, saline 3%; IV, intravenous fluids*.

Upon 14 h of fluid replacement, he was started on normal saline in order to maintain his serum sodium at 150 mmol/L, so as not to reduce the serum sodium levels of more than 10 mmol/L over 24 h. Despite on normal saline, the serum sodium levels continued to plummet to 146 mmol/L, hence a decision was made to infuse 200cc of 3% saline over 4 h. This resulted in a desired 3 mmol increase in the serum sodium. His baseline blood results prior to admission and on presentation along with his intravenous fluid regimens are shown in Table 1. The patient was allowed to consume oral fluids as per tolerated.

However, by day 2 of admission, the serum sodium levels increased to 150 mmol/L again, thus it was decided to reduce the serum sodium levels further with intravenous crystalloids and frequent biochemical monitoring. The aim was a controlled serum sodium reduction of 10 mmol/L/day as well as adequate hydration to improve the serum urea and creatinine levels (Table 2). The patient was started with alternate 500 mls normal saline and normal saline dextrose 5% infusion every 3 hourly. A decrement of 8 mmol/L was achieved within 17 h, hence the fluid regime was further adjusted to 500cc of normal saline 4 hourly for the next 2 days until his sodium levels were static.

**Table 2:** Biochemical parameters from day 2 till 7 of hospital stay.

**Parameters**	**Normal range**	**D2**		**D3**			**D4**	**D5**	**D6**	**D7**
		**07:00 h**	**16:00 h**	**00:00 h**	**08:00 h**	**20:00 h**	**08:00 h**	**08:00 h**	**08:00 h**	**13:00 h**
Haematocrit	40.1–50.6%	51.2			51.5			48.2		50.6
Hemoglobin	13.5–17.4 g/dL	16.1			16.2			15.4		17.0
Na	135–145 mmol/L	150	145	142	146	145	145	142	140	141
K	3.5–4.5 mmol/L	4.1	4.1	3.8	3.8	3.2	3.3	3.0	3.2	3.8
Urea	3.2–7.4 mmol/L	37.9	32.8	26.9	23.5	19.4	15.9	10.4	7.4	7.4
Creatinine	63.6–110.5 umol/L	171.3	144.6	114.3	113.0	85.9	83.2	62.1	58.8	64.1
Albumin	35–50 g/L				30					
Serum osmolarity	mOsm/L	344	351	325	325		317	309		
Urine osmolarity	mOsm/L	554	579	549	524		501			
Urine sodium	mmol/L	52	31	44	72		102			
Fluids given		Alternate 500cc NSD5% and 500cc NS 3 hourly			500 cc NS 4 hourly			Stop IV Fluids	500cc NSD5% 8 hourly	
Daily urine output		700cc			880cc					
Daily fluid balance		+3019cc			+1706cc					

*NS, normal saline; NSD5%, normal saline dextrose 5%; IV, intravenous fluids*.

By day 5, the intravenous fluid infusion was temporarily withheld as the patient's serum sodium had normalized but was restarted again on day 6 with normal saline dextrose 5% with additional potassium supplement as his serum potassium was still low and patient was not taking orally that well.

His oral intake subsequently improved and he was discharged well uneventfully by day 7 of admission without any complications. Unfortunately, the patient was once again lost to follow up when he did not turn up for his subsequent clinic appointment.

## Discussion

Sodium levels in the body are tightly regulated via the body's own intricate homeostatic mechanism that corrects any fluctuations in the blood volume, blood pressure, and serum osmolarity (1). Normal physiology dictates that an increasing serum sodium level leads to increased vasopressin secretion, in addition to increased thirst sensation in a healthy individual.

However, these intrinsic compensatory mechanisms may fail resulting in hypernatremia, which is defined as serum sodium concentration exceeding 145 mmol/L (2). When the serum sodium is more than 150 mmol/L, it is then labeled as severe hypernatremia (3). Hypernatremia can be further classified according to the patient's fluid status, namely hypovolaemic, euvolaemic, and hypervolaemic, respectively. This is important as the treatment of hypernatremia varies accordingly.

Commonly, hypernatremia is usually caused by hypovolaemia due to water loss, either by increased loss or reduced intake. Rarely it can be due to sodium gain where notably there have been several case reports of salt-related poisoning (4, 5).

This patient's hypernatremic status may be a result of both causes as he was cut off from water access and hence dehydrated, as well as increased salt ingestion through his urophagia. In patients with normal kidney function, the body is able to maintain the urine osmolality within a normal range. Once dehydration sets in, the urine will be maximally concentrated, and the urine osmolality will be increased. Since we do not have his urine osmolality at presentation, we estimated that his urine osmolality would probably be at least two to three-fold higher than the serum osmolality, an expected urine osmolality seen in dehydrated individuals. Essentially it is akin to drinking hypertonic saline which has an osmolality of 1,027 mOsm/L. Similar findings may also be seen in those who were stranded at sea and had to naively resort to drinking sea water (~1,000 mOsmol/L) to quench their unremitting thirst.

Another important contributor to the hypernatremia was the high serum urea found in the patient as a result of drinking his own urine. It is believed that the high urea resulted in osmotic diuresis giving rise to hypovolemic hypernatremic state similar to the use of mannitol in inducing osmotic diuresis (6).

It has been reported in some critically ill patients whereby urea is being used for the treatment of hyponatraemia in the syndrome of inappropriate secretion of antidiuretic hormone in order to provoke osmotic diuresis, and was recently described to be complicated by the occurrence of hypernatraemia.

When serum sodium starts to rise in a patient who is in a catabolic state, a high urea in the urine that exceeds the amount of urinary sodium and potassium should give a clue to the presence of osmotic urea diuresis. In this case scenario action should be taken to reduce the urea load for the patient and if this is not feasible then adequate free water should be given to avoid a further rise in serum sodium.

Osmotic diuresis due to high urea is often not recognized in hypernatremia since the higher urine osmolality compared to the serum osmolality could mean that the kidneys are trying to retain water.

To the best of our knowledge this is the first reported case of hypernatremia caused by dehydration and high urea osmotic diuresis triggered by drinking his own urine (urophagia). Historically urophagia, or ingestion of human or animal urine, is traditionally associated with medicinal purposes, despite there are no proven benefits (7). There have been media reports on individuals resorting to drinking their own urine as a survival technique as they are cut off from access to food and water (8–10).

## Conclusion

Hypernatremia from drinking one's own urine is triggered by several mechanisms, the most profound is the presence of high serum urea that drives significant osmotic diuresis leading to hypovolemia and subsequent hypernatremia.

## Data Availability Statement

The raw data supporting the conclusions of this article will be made available by the authors, without undue reservation.

## Author Contributions

Article conception: NK and WK. Data collection and drafting the article: WK and NS. Critical revision of the article and final approval of the article to be published: WK, NS, and NK. All authors were responsibility of the inpatient management of the patient in the case report. All authors contributed to the article and approved the submitted version.

## Conflict of Interest

The authors declare that the research was conducted in the absence of any commercial or financial relationships that could be construed as a potential conflict of interest.

## Publisher's Note

All claims expressed in this article are solely those of the authors and do not necessarily represent those of their affiliated organizations, or those of the publisher, the editors and the reviewers. Any product that may be evaluated in this article, or claim that may be made by its manufacturer, is not guaranteed or endorsed by the publisher.
